# Photoresponsive Luminescent Polymeric Hydrogels for Reversible Information Encryption and Decryption

**DOI:** 10.1002/advs.201901529

**Published:** 2019-09-12

**Authors:** Zhiqiang Li, Hongzhong Chen, Bin Li, Yanmiao Xie, Xiaoli Gong, Xiao Liu, Huanrong Li, Yanli Zhao

**Affiliations:** ^1^ National‐Local Joint Engineering Laboratory for Energy Conservation in Chemical Process Integration and Resources Utilization Tianjin Key Laboratory of Chemical Process Safety School of Chemical Engineering and Technology Hebei University of Technology Guangrong Dao 8, Hongqiao District Tianjin 300130 P. R. China; ^2^ Division of Chemistry and Biological Chemistry School of Physical and Mathematical Sciences Nanyang Technological University 21 Nanyang Link Singapore 637371 Singapore; ^3^ College of Computer Nankai University No. 38 Tongyan Road, Jinnan District Tianjin 300350 P. R. China

**Keywords:** information encryption and decryption, luminescence, photoresponsive materials, polymeric hydrogels, tunable emission

## Abstract

Conventional luminescent information is usually visible under either ambient or UV light, hampering their potential application in smart confidential information protection. In order to address this challenge, herein, light‐triggered luminescence ON‐OFF switchable hybrid hydrogels are successfully constructed through in situ copolymerization of acrylamide, lanthanide complex, and diarylethene photochromic unit. The open‐close behavior of the diarylethene ring in the polymer could be controlled by UV and visible light irradiation, where the close form of the ring features fluorescence resonance energy transfer with the lanthanide complex. The hydrogel‐based blocks with tunable emission colors are then employed to construct 3D information codes, which can be read out under a 254 nm UV lamp. The exposure to 300 nm UV light leads to the luminescence quenching of the hydrogels, thus erasing the encoded information. Under visible light (>450 nm) irradiation, the luminescence is recovered to make the confidential information readable again. Thus, by simply alternating the exposure to UV and visible lights, the luminescence signals could become invisible and visible reversibly, allowing for reversible multiple information encryption and decryption.

## Graphical abstract

**Light‐triggered luminescence ON‐OFF switchable hybrid hydrogels** are synthesized through in situ copolymerization. The hydrogel‐based blocks with tunable emission colors are then employed to construct 3D information codes, allowing for reversible multiple information encryption and decryption.

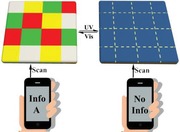

Photoluminescent soft materials have been widely applied in sensing, display devices, and organic light‐emitting diodes,^1^ and also have received great attention toward security protection applications in information storage, date recording and encryption.^2^ In particular, luminescent hydrogel‐based 3D codes prepared in environmentally friendly process could not only increase the information density per unit area but also be employed as wearable or biological anti‐counterfeiting materials.^3^ On the other hand, the information recorded directly in these materials is usually visible under either ambient or UV light, which would hamper their practical applications in confidential information protection because these anti‐counterfeiting labels could be easily mimicked.^4^

In this context, smart luminescent materials that can perceive the surrounding stimuli and respond to them should be ideal for confidential information protection.^5^ Under external stimuli, the luminescent outputs of these materials can be precisely modulated, preventing the information from being stolen or mimicked.^6^, ^7^ Stimulus‐responsive luminescent materials that rely on constant addition of chemicals have been developed for information encryption and decryption.^8^ Since these methods require invasive stimuli, it may be difficult for consumers without professional chemistry knowledge to handle the encoded information by adding chemicals. Therefore, it is highly desirable to develop alternative switchable luminescent materials with confidential encryption property capable of being easily operated in a noninvasive manner, where the security codes are initially invisible and become visible under specific external stimuli. In this way, reversible information encryption and decryption could be achieved. Light irradiation is an appealing external stimulus because it provides clean, spatiotemporal, and noninvasive control on the operation with high precision,^9^ showing greater convenience in activating or erasing the code information as compared to other chemical stimuli.^10^

In terms of emitting sources, lanthanide complexes are excellent emitting centers because of their intriguing optical properties, such as narrow emission bands, large Stokes shift, high luminescent efficiency, and long luminescence lifetime.^7^, ^11^ To the best of our knowledge, however, achieving ON‐OFF photoswitchable lanthanide‐containing luminescent hydrogels is still challenging. This is mainly because that the —OH vibration of water molecule can quench the excited state of lanthanide significantly, thereby making it even more difficult to further modulate the emitting behavior of lanthanide in aqueous condition.^12^, ^13^ By controlling the interconversion of photochromic compounds between two states with different spectroscopic properties to achieve the modulation of fluorescence resonance energy transfer (FRET), electron transfer or charge transfer process, integrating photoswitchable molecules and lanthanide ions could be a feasible strategy to develop luminescent ON‐OFF switchable organogels.^14^, ^15^ This strategy may provide an appealing approach for rational design of photoswitchable lanthanide‐containing hydrogels.

Herein, we developed an approach to realize reversible confidential information encryption and decryption based on photoresponsive luminescence ON‐OFF of robust lanthanide‐containing hydrogels. The hydrogel was constructed by in situ copolymerization of acrylamide (AAm) monomers with a rational incorporation of diarylethene photochromophore and luminescent lanthanide center (**Scheme** 1). Diarylethene as a promising photoswitchable molecule in light‐triggered systems^16^ could undergo the open and close isomerization under alternating UV and visible light irradiation, which was employed as the photoresponsive unit in the hydrogel.^17^ To transfer the isomerization of diarylethene unit to the luminescence switch of hydrogels, we employed a lanthanide complex to not only maintain excellent luminescent properties in aqueous condition but also achieve spectral overlapping with the diarylethene unit as the emitting center. The photochromic FRET between Ln^3+^ and diarylethene is typically governed by the conformation of diarylethene.^14^, ^18^ Since the absorption of the open form diarylethene has no spectral overlapping with the emission of Ln^3+^, the hydrogel monolith exhibits characteristic emission of Ln^3+^ and the information stored in hydrogel codes can be read out. On the other hand, the absorption of the close form diarylethene shows perfect spectral overlapping with the emission of Ln^3+^, leading to the luminescence quenching of Ln^3+^ on account of the activation of the FRET (**Scheme** 2). In this case, the information stored in hydrogel codes becomes invisible, resembling the addition of an extra lock to the security code. In particular, combining wide absorbance band (450–700 nm) of the close form diarylethene with narrow emission band of green (Tb^3+^) and/or red (Eu^3+^) lanthanide emitting centers allows for simultaneous occurrence of FRET between them. Therefore, the emission color of the hydrogels could be fine‐tuned from green through yellow to red by altering the molar ratio of Eu^3+^ and Tb^3+^ ions in the polymer, providing a large analogue matrix of switchable optical outputs.^19^ Hence, the open and close isomerization of the diarylethene unit regulates the inactivation and activation of the FRET process, resulting in a reversible luminescence ON‐OFF of lanthanide emitting centers in the hydrogel capable of conducting reversible multiple information encryption and decryption.

**Scheme 1:**
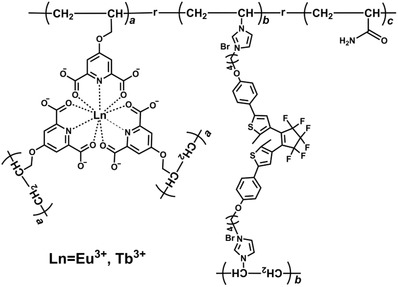
Chemical structure of the polymer consisting of AAm, lanthanide complex, and diarylethene photochromic unit for the formation of hydrogels.

**Scheme 2:**
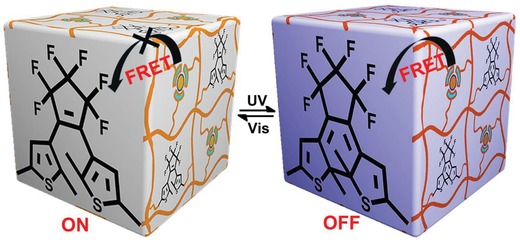
Illustrated luminescent ON‐OFF photoswitchable behavior of lanthanide‐containing hydrogels under alternating UV and visible light irradiation.

Lanthanide/2,6‐pyridinedicarboxylic acid complex (Ln(PDA)_3_) was functionalized with a propenyl unit to prepare the emitting source **Ln·L_3_** according to our previously reported method.^20^ Vinyl imidazolium salt modified diarylethene (**1**) with satisfied water solubility was also prepared (Figures S1–S6, Supporting Information). Transparent and self‐standing luminescent hydrogels were subsequently prepared through in situ copolymerization of AAm, **Ln·L_3_** and **1** (see the Experimental Section). The mechanical properties of the hydrogels were characterized by tensile stress–strain and rheological testing. Upon stretching, the hydrogel sheets underwent a continuous extension, withstanding a fracture stress up to 80 kPa with the elongation of more than five times longer than its original length (**Figure** 1A). Eu^3+^‐ and Tb^3+^‐containing hydrogels as well as Eu^3+^/Tb^3+^ codoped hydrogel (Tb^3+^/Eu^3+^ = 1:1) exhibited similar tensile strength, indicating that the type and the molar ratio of lanthanide ions have a negligible impact on the mechanical behavior of the resultant hydrogels (Figure S7, Supporting Information). Rheological tests on these hydrogels showed that the elastic behavior was dominant in the applied frequency range. The storage modulus (*G*′) value was larger than the loss modulus (*G*″) value, which is the characteristic of a stable gel phase (Figure 1B and Figure S8, Supporting Information). Improved healing properties are originated from supramolecular interactions, such as hydrogen bonding, macrocyclic host–guest, and metal–ligand interactions.^21^ In light of the existence of hydrogen bonds between PAAm and lanthanide complex cross‐linker in the hydrogels, the self‐healing behavior was expected in our system. The strain‐dependent oscillatory rheological experiments (Figure 1C) and step‐strain rheological measurements (Figure 1D) show that the gel state could be recovered quickly after treating it with a large amplitude oscillatory force, providing strong evidence that the hydrogels possess the self‐healing (i.e., cutting and readhering with the same hydrogels) or heterohealing (i.e., cutting and readhering of hydrogels with different lanthanide emitting sources) ability (Figure S9, Supporting Information). Tensile testing exhibits that the self‐healed hydrogel still displays comparable fracture stress with the original one, and could be stretched to more than 400% of its original length (Figure 1A). These properties allow the freshly cut hydrogels to be programmed and adjusted as optical matrix on a substrate. Direct morphological information of the supramolecular hydrogels was obtained by scanning electron microscopy (SEM). The SEM images reveal uniform nature of the hybrid hydrogels, probably attributed to relatively high concentration of AAm in the hydrogels. After freeze‐drying, the xerogels exhibited typical porous structures (Figure S10, Supporting Information).

**Figure 1:**
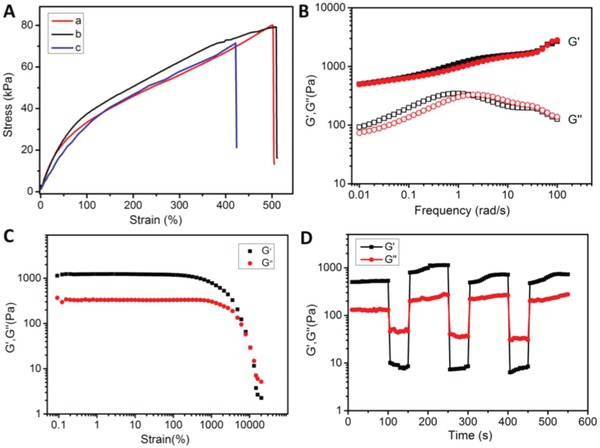
A) Tensile stress–strain curves of the Eu^3+^‐containing hydrogel (a) before and (b) after UV light irradiation, and (c) freshly cut Eu^3+^‐containing hydrogel after self‐healing for 10 h. B) Frequency (ω) sweep tests at ω = 0.01–100 rad s^−1^ and strain (γ) = 1.0% for Eu^3+^‐containing hydrogel before (black squares) and after (red circles) irradiation with UV at 25 °C. C) Strain sweep tests of Eu^3+^‐containing hydrogel at γ = 0.1–100 000% and ω = 1.0 Hz. D) Continuous step strain tests of Eu^3+^‐containing hydrogel at γ = 0.1 and 50 000% with ω = 1.0 Hz.

We then investigated the photoluminescent property of the obtained hydrogels. Taking Eu^3+^‐containing hydrogel as the example, the excitation spectrum by monitoring the ^5^D_0_→^7^F_2_ transition displays a broad excitation band in the range of 250–320 nm, resulting from the π–π* transitions of PDA unit (Figure S11, Supporting Information).^12^, ^22^ Negligible intensity of the intra‐4f^6^ transition in the excitation spectrum indicates that an energy transfer occurs from the ligand to Eu^3+^ ion. The corresponding emission spectrum consists of five sharp emission bands at 582, 594, 615, 650, and 694 nm, attributing to the ^5^D_0_→^7^F*_J_* (*J* = 0–4) transitions.^23^ The primary band at 615 nm is assigned to the ^5^D_0_→^7^F_2_ transition and responsible for the red emission. Thus, emission color could be tailored in a broad spectrum range from green through yellow to red by altering the molar ratio of the two lanthanide chromophores in the hydrogels (**Figure** 2). The overall quantum yield (Φ), excited state lifetime (τ), and coordinated water number (*q*) of Ln^3+^ in the hydrogels were also determined (Figures S12 and S13, Supporting Information) and listed in Table S1 (Supporting Information), indicating their sufficient brightness for applications.

**Figure 2:**
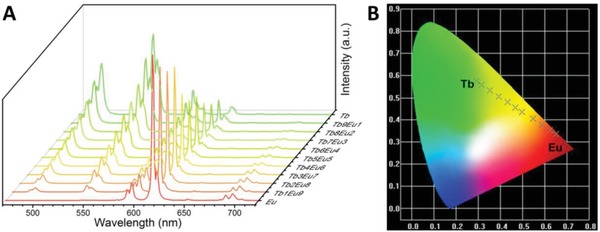
A) Luminescence emission spectra of the hydrogels with varied Eu^3+^/Tb^3+^ molar ratios (λ_ex_ = 280 nm). Tb^3+^/Eu^3+^ = 10:0, 9:1, 8:2, 7:3, 6:4, 5:5, 4:6, 3:7, 2:8, 1:9, 0:10; B) The corresponding CIE 1931 chromaticity diagram with varied Eu^3+^/Tb^3+^ molar ratios.

The photoresponsive luminescence behavior of the hydrogels as a result of the diarylethene isomerization was investigated. Before conjugating with the **Ln·L_3_** component, individual **1** successfully underwent reversible open and close isomerization upon alternating exposure to UV and visible light. As shown in **Figure** 3, the UV–vis spectrum of the open form **1** (OF‐**1**) showed a strong absorption at 298 nm. When the solution of OF‐**1** was irradiated with UV light at 300 nm, the peak at 298 nm gradually decreased and new peaks appeared at 350 and 592 nm with an isosbestic point at 325 nm. These changes reached to photostationary state in 60 s, leading to an obvious color change of the solution from colorless to dark blue (Figure 3, inset). A combination of these phenomena indicates the transition from OF‐**1** to the close form **1** (CF‐**1**). To further clarify the isomerization behavior of compound **1**, ^1^H NMR spectra of compound **1** in DMSO were recorded to avoid the signal broadening in aqueous medium (**Figure** 4). Upon 300 nm light irradiation, the thiophene protons (H_b_) underwent drastic upfield shift from 7.38 to 6.89 ppm, while the aromatic protons (H_c_ and H_d_) showed downfield shifts (Δδ = 0.15 ppm for H_c_ and 0.05 ppm for H_d_). The methyl protons (H_a_) also showed downfield shift from 1.95 to 2.06 ppm. A slight downfield shift was observed in the methylene protons (H_e_) near the benzene unit. All these shifts were complete, and no residual signals were observed in the original chemical shifts, indicating quantitative transition from OF‐**1** to CF‐**1** after 300 nm light irradiation. Interestingly, a complete recovery of the original UV–vis spectrum of OF‐**1** was achieved upon subsequent irradiation of CF‐**1** with visible light (λ > 450 nm) in 60 s, accompanied with the color change from dark‐blue solution back to colorless.

**Figure 3:**
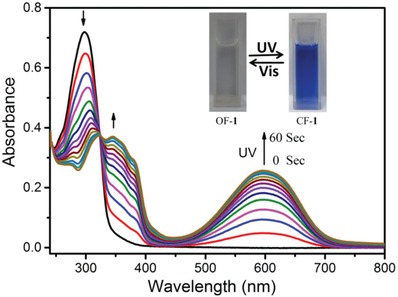
UV–vis spectral changes of **1** aqueous solution (2.0 × 10^−5^ m) upon irradiation with 300 nm UV light for up to 60 s. Inset: corresponding photographic images of **1** aqueous solution upon alternating UV and visible light irradiation.

**Figure 4:**
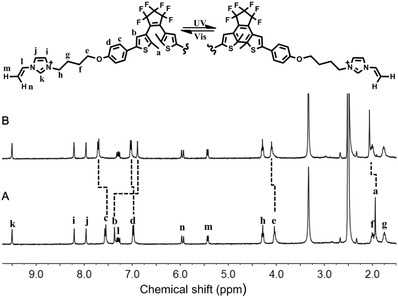
A,B) Partial ^1^H NMR spectra (DMSO, 400 MHz, 25 °C) of compound **1** before and after irradiated at 300 nm for 10 min.

In the as‐prepared hydrogels, the diarylethene unit showed the absorption below 400 nm, as it was in the open configuration and there was no spectral overlapping between the emission of **Ln·L_3_** component and the absorption of diarylethene component. Therefore, no FRET occurred, and the hydrogels exhibited the characteristic color and brightness of Ln^3+^ complexes under 254 nm light (**Figures** 2 and 5A,B). After exposing the luminescent hydrogels to 300 nm UV light, the diarylethene unit switched from open to close form with a new absorption band in the range of 450–700 nm. This absorption range overlaps with the luminescence emission range of the **Ln·L_3_** components, and therefore efficient photochromic FRET from Ln^3+^ to the close form of diarylethene unit in the hydrogels was expected (Figure 5A). Taking Eu^3+^‐containing hydrogel as an example, the luminescence intensity of the hydrogel was gradually quenched to reach the equilibrium after irradiated with 300 nm UV light for 60 s (Figure 5B). At this point, the luminescence intensity was quenched by 91.8% with concomitant decreases of the decay from 872 to 68 µs and quantum yield from 12.29 to 0.62%. The photochromic FRET efficiency in the hydrogel was calculated to be 92% according to the reported method.^24^ Tensile stress and rheological testing of the hydrogel after irradiation with 300 nm UV (Figure 1A,C and Figure S8, Supporting Information) showed no obvious mechanical strength loss as compared with those of the original hydrogel. This observation indicates that the luminescence ON‐OFF photoswitch process would not affect the mechanical behavior of the hydrogel, as the open and close isomerization of diarylethene unit does not destroy the hydrogel network. It should be noticed that diarylethene derivatives usually do not undergo reversible photoisomerization spontaneously, and the close form could be maintained under daylight for a long period of time.^25^ The stability of CF‐**1** solution and corresponding hydrogel were measured for one month, and no obvious automatic recovery in UV–vis spectra or luminescence emission spectra was observed during this period (Figures S14 and S15, Supporting Information). Moreover, the luminescence intensity of the resultant dark blue hydrogel could be restored to the initial state upon >450 nm visible light irradiation for 60 s, arising from the regeneration of OF‐**1** (Figure S16, Supporting Information). Notably, this light‐driven luminescence switching cycle could be repeated without fatigue for at least ten times (Figure 5C). High‐efficiency light‐triggered photochromic FRET process was also observed in Tb^3+^‐containing and Tb^3+^/Eu^3+^ codoped hydrogels under UV light irradiation (Figures S17 and S18, Supporting Information), through which photoreversible luminescence ON‐OFF of robust lanthanide‐containing hydrogels with wide emitting range was achieved.

**Figure 5:**
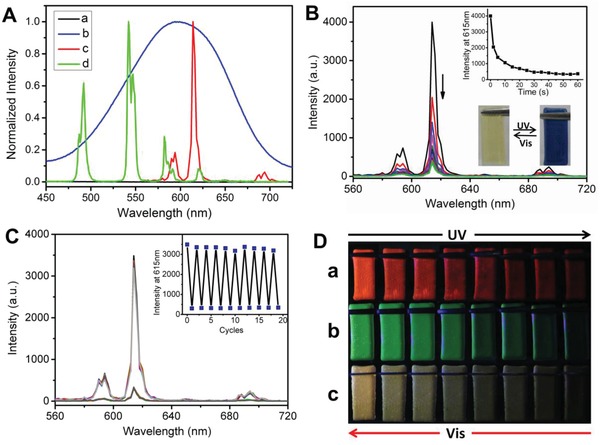
A) Normalized absorption spectra of (a) OF‐**1** and (b) CF‐**1**, as well as emission spectra of (c) Eu^3+^‐containing and (d) Tb^3+^‐containing hydrogels. B) Luminescence emission spectra (λ_ex_ = 280 nm) and emission intensity changes at 615 nm (upper inset) for Eu^3+^‐containing hydrogel upon 300 nm UV light irradiation. The lower inset shows the photographs of Eu^3+^‐containing hydrogel under daylight upon alternating irradiation with 300 nm UV light and visible light (λ > 450 nm). C) Luminescence emission spectra and intensity changes at 615 nm (inset) for Eu^3+^‐containing hydrogel upon repeatedly alternating irradiation with UV and visible light. D) Luminescence emission color changes of (a) Eu^3+^‐containing, (b) Tb^3+^‐containing, and (c) Tb^3+^/Eu^3+^ codoped (Tb^3+^/Eu^3+^ = 1:1) hydrogels under 254 nm light upon alternating irradiation with 300 nm UV and visible light (λ > 450 nm). The size of the hydrogel blocks is 2.5 cm × 1 cm × 0.2 cm.

High‐photoluminescence quantum yield, narrow emission spectra, wide color gamut, and especially reversible luminescence ON‐OFF switch behavior of the hydrogels encouraged us to further explore their potential application in constructing smart data encryption systems. Hydrogel blocks without luminescence (nonhydrogel) were also prepared by the copolymerization of acrylamide and **1** in the absence of **Ln·L_3_** to provide a uniform background and enrich the optical information matrix. The red hydrogel (Eu^3+^‐containing hydrogel), green hydrogel (Tb^3+^‐containing hydrogel), yellow hydrogel (codoped hydrogel), and nonhydrogel were then placed on a black substrate and contacted with each other to construct an adhesion‐based luminescent array according to **Code A**. Under daylight, no luminescence was observed, and the information stored in the code cannot be recognized by the application software in a smartphone (Movie S1, Supporting Information). Under 254 nm UV lamp, the luminescent matrix appeared and could be recognized by the software easily, allowing readout of the coding information (**Figure** 6A,B). The main absorption band of OF‐**1** appears from 260 to 340 nm (Figure 3). As a result, the light‐triggered isomerization from open form to close form is relatively slow under 254 nm UV lamp, and the luminescent pattern is recognizable during the first several minutes, providing enough time for reading out the coding information. For the sake of easy illustration, we programed the information and loaded it to a specific website (Movie S2, Supporting Information). When irradiated with 300 nm UV light, the luminescence of the gel blocks was quenched, accompanied with the disappearance of the luminescent pattern. As a result, the coding information became invisible and cannot be read out (Figure 6C,D and Movie S3, Supporting Information), through which encryption was realized. The quenched pattern was also very stable under daylight and still unreadable even after one month (Figure S19 and Movie S4, Supporting Information). The erased pattern could only be decrypted and completely recovered when needed, upon further irradiating by visible light with special wavelength (>450 nm). The photocontrolled information encryption and decryption process showed excellent fatigue resistance and still remained unaffected even after ten consecutive switching cycles (Movie S5, Supporting Information). The encoded information within the 3D code‐producing array could be revised by changing the pattern of the array. For instance, **Code A** was transformed to a new array (**Code B**) by changing the positions of hydrogel blocks on the same substrate. Under UV light irradiation, the newly encoded information could be read out easily by the smartphone (Figure 6E,F and Movie S6, Supporting Information). Diverse patterns could be assembled using this reassembly approach.

**Figure 6:**
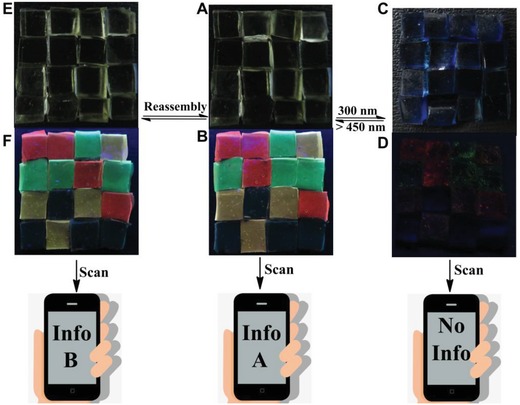
A,C) Photographs of a pattern (**Code A**) made up from the assembly of red hydrogel, green hydrogel, yellow hydrogel, and nonhydrogel on a black substrate under daylight before and after irradiation with 300 nm UV light. B,D) Under these conditions, the information could be read out or masked. E,F) Photographs showing the transformation of **Code A** into **Code B** under daylight and 254 nm UV light by means of a reassembly strategy. Hydrogels were all prepared on the same substrate. Photographs of (A), (C), and (E) were taken under daylight, and photographs of (B), (D), and (F) were taken under 254 nm UV light. The size of each hydrogel block in the code pattern is 0.5 cm × 0.5 cm × 0.2 cm.

While we here just took Tb^3+^/Eu^3+^ (1:1) codoped hydrogel as an example, the emission color of the hydrogels could be finely tuned from green to red by altering the molar ratio of Eu^3+^ and Tb^3+^ ions, and the size and morphology of the gel blocks could be programmed according to the real need. These two advantages together with the reassembly approach would greatly enrich optical outputs and provide limitless number of information‐rich codes. The encoded data on programmable optical matrix with light triggered invisible/visible reversible coding information are unable to be mimicked and stolen. While the information protection based on chemical means has been reported, this light‐controlled reversible information encryption and decryption system could be effectively concealed and read out by simply alternating the irradiation with UV and visible light, making it suitable for the next‐generation of intelligent security protection.

In conclusion, we have successfully constructed lanthanide‐containing hydrogels with photoresponsive luminescence ON‐OFF switch behavior through controlling the FRET process between lanthanide and photochromic units, arose from light‐induced open and close isomerization of the diarylethene derivative. The emission colors of the resulted hydrogels could be precisely tailored in a wide spectrum range by using different lanthanide ions with different molar ratios. The hydrogel blocks with various luminescence emission colors (i.e., red, green, yellow, and colorless) were assembled into 3D code arrays for confidential information protection. Upon the irradiation with 300 nm UV light, the luminescent pattern was masked as a result of the open to close form transformation of the diarylethene unit, while further visible light irradiation could completely recover the luminescent pattern. The encoded information could be easily read out under commercial UV lamp. Thus, this smart luminescent system could be employed for reversible confidential information encryption and decryption through simple light irradiation, which provides a new avenue for the development of next‐generation smart anti‐counterfeiting materials and devices.

## Experimental Section

Experimental details including the preparation and characterization could be found in the Supporting Information.

## Conflict of Interest

The authors declare no conflict of interest.

## Supporting information

Supplementary

Supplementary

Supplementary

Supplementary

Supplementary

Supplementary

Supplementary
