# Supplementary material for: Photoresponsive Luminescent Polymeric Hydrogels for Reversible Information Encryption and Decryption
Source: Adv Sci (Weinh). 2019 Sep 12;6(21):1901529. doi: 10.1002/advs.201901529 (PMC6839628; doi:10.1002/advs.201901529)
Supplement: Supplementary file 1 — Supplementary [file ADVS-6-1901529-s002.pdf]

Copyright WILEY-VCH Verlag GmbH & Co. KGaA, 69469 Weinheim, Germany, 2019.

Supporting Information

for *Adv. Sci.,* DOI: 10.1002/advs.201901529

Photoresponsive Luminescent Polymeric Hydrogels for Reversible Information Encryption and Decryption

Zhiqiang Li, Hongzhong Chen, Bin Li, Yanmiao Xie, Xiaoli Gong, Xiao Liu, Huanrong Li, and Yanli Zhao**

Supporting Information

Materials and methods

All chemicals were commercially available unless noted otherwise. NMR spectra were recorded on a Bruker 400 instrument. Tensile properties were determined on CMT6104. The measurements were carried out at a crosshead speed of 100 mm/min. Rheological tests of hydrogels were carried out by using an Anton Paar model MCR-301 rheometer, with a 25 mm diameter parallel plate attached to a transducer. After placing the sample, the upper plate was slowly lowered until a gap of size 0.5 mm was reached. The frequency sweep was performed over the frequency range of ω = 0.01 - 100 rad s^{-1} at a fixed strain of 1.0%. The dynamic strain sweep was carried out at γ = 0.1 - 100000% and ω = 1.0 Hz to determine the linear viscoelasticity region. Continuous step strain tests were measured at γ = 0.1 and 50000% with ω = 1.0 Hz. The steady-state luminescence spectra were measured on an Edinburgh Instruments FS920P near-infrared spectrometer, with a 450 W xenon lamp as the steady-state excitation source, a double excitation monochromator (1800 lines·mm^{-1}), an emission monochromator (600 lines·mm^{-1}), and a semiconductor cooled Hamamatsu RMP928 photomultiplier tube. The 300 nm UV light irradiation experiment was carried out under a 500W Hg lamp with 300 nm optical filter, and the visible light irradiation experiment was carried out using a CEL HXF300 xenon lamp with >450nm cutoff filter. The photo and decryption/encryption application were taken under a ZF-7A lamp (254 nm, 8 W). The software used to read the signals was written according to the emission range (from green to red) of the hydrogels, which could recognize square luminescent array. This algorithm firstly finds the outer contour of all hydrogels in the binarized pre-processed image and divides the interior of the contour into squares to represent each hydrogel. It is obvious that the color of the largest inscribed rectangle can dominantly represent the color of this hydrogel. Because the H channel in the HSV image shows the pixel color, the hydrogel color is finally recognized by computing the average value of the H channel of all the pixels in the largest inscribed rectangle.

Experimental section

Preparation of compound 3:

4-Hydroxyphenylboronicacidpinacolester (220.1 mg, 1 mmol), 1,4-dibromobutane (1079.6 mg, 5 mmol), and K_2CO_3 (690 mg, 5 mmol) were added into acetonitrile (30 mL) with stirring. The mixture was heated at 70°C under N_2 atmosphere for 24 h. After cooling down to room temperature, the reaction mixture was filtered and the residue was washed with CH_2Cl_2. Then, the filtrate was concentrated under a reduced pressure. The residue was dissolved by CH_2Cl_2 (100 mL) and washed twice with saturated NaCl solution. The organic phase was dried over anhydrous Na_2SO_4, and then concentrated. The crude product was purified by column chromatography over silica gel (eluent: 20:1 PE/EA), and compound **3** was obtained as white powder in 92% yield. ^{1}H NMR (400 MHz, $CDCl_3$): δ 7.76 (d, J = 8.5 Hz, 2H), 6.90 (d, J = 8.5 Hz, 2H), 4.04 (t, J = 6.0 Hz, 2H), 3.51 (t, J = 6.6 Hz, 2H), 2.10 (m, 2H), 1.97 (m, 2H), 1.35 (s, 12H). HRMS $[M + Na]^+$ calcd for $C_{16}H_{24}BBrO_3Na^+$ 377.0900; found: 377.0899; Anal Cald for $C_{16}H_{24}BBrO_3$: C, 54.12; H, 6.81; Found: C, 54.08; H, 6.75.

Preparation of compound 2:

To a 100 mL two-neck round flask, 3,3'-(3,3,4,4,5,5-hexafluoro-1-cyclopentene-1,2-diyl) bis[5-bromo-2-methylthiophene][26] (210 mg, 0.40 mmol), compound **3** (360 mg, 1mmol), Na_2CO_3 (680 mg, 6.4 mmol), water (4 mL), and 1,4-dioxane (30 mL) were added under N_2 atmosphere. Then, $Pd(PPh_3)_4$ (70 mg, 0.06 mmol) was added. The reaction mixture was refluxed at 90 °C in dark for 24 hours. After cooling down to room temperature, the solvent was removed under vacuum, and the residue was extracted by dichloromethane. Dichloromethane was removed under reduced pressure, and the residue was purified on a silica gel column using EA/PE (1:10) as the eluent to give compound **2** as a pale yellow solid (yield: 70%). ^{1}H NMR (400 MHz, $CDCl_3$): δ 7.47 (d, J = 8.7 Hz, 4H), 7.17 (s, 2H), 6.91 (d, J = 8.7 Hz, 4H), 4.04 (t, J = 6.0 Hz, 4H), 3.52 (t, J = 6.5 Hz, 4H), 2.10 (dd, J = 14.8, 6.5 Hz, 4H), 1.99 (dd, J = 13.6, 5.1 Hz, 4H), 1.96 (s, 6H). HRMS $[M]^+$ calcd for $C_{35}H_{32}Br_2F_6O_2S_2^+$ 822.0094; found: 822.0087; Anal Cald for $C_{35}H_{32}Br_2F_6O_2S_2$: C, 51.11; H, 3.92; Found: C, 51.09; H, 3.88.

Preparation of compound 1:

Compound **2** (123.4 mg, 0.15 mmol) was dissolved in acetonitrile (10 mL), and then 1-vinylimidazole (42.4 mg, 0.45 mmol) was added. The reaction mixture was stirred at 80 °C for 12 h. After cooling down to room temperature, the obtained white precipitate was collected by centrifugation and washed with diethyl ether for 3 times to afford the desired product OF-**1** in 90% yield. ^{1}H NMR (400 MHz, DMSO-d$_6$): δ 9.52 (s, 2H), 8.21 (s, 2H), 7.97 (s, 2H), 7.56 (d, J = 8.7 Hz, 4H), 7.37 (s, 2H), 7.30 (dd, J = 15.6, 8.7 Hz, 2H), 6.98 (d, J = 8.7 Hz, 4H), 5.96 (dd, J = 15.6, 2.3 Hz, 2H), 5.43 (dd, J = 8.7, 2.3 Hz, 2H), 4.29 (t, J = 7.1 Hz, 4H), 4.04 (t, J = 6.1 Hz, 4H), 2.00 (dd, J = 14.6, 7.4 Hz, 4H), 1.95 (s, 6H), 1.81-1.69 (m, 4H). HRMS $[M-2Br]^{2+}$ calcd for $C_{45}H_{44}F_6N_4O_2S_2^{2+}$ 425.1405; found: 425.1403; Anal Cald for $C_{45}H_{44}Br_2F_6N_4O_2S_2$: C, 53.47; H, 4.39; N, 5.54; Found: C, 53.39; H, 4.30; N, 5.51.

Preparation of hybrid hydrogels:

Acrylamide (414 mg, 5.8 mmol, 0.31 mL) was dissolved in water (1.69 mL) to prepare acrylamide stock solution. Then, **Eu·L$_3$** (11.6mg, 0.0125mmol) was added, followed by the addition of compound **1** (12.75 mg, 0.0125 mmol) into the mixture. Next, azodiisobutyronitrile (AIBN, 5 mg, 0.03 mmol) was added to DMSO solution (0.5 mL). The two solutions were bubbled with nitrogen for 10 min to eliminate the dissolved oxygen. Finally, the two solutions were mixed and bubbled with nitrogen for another 10 min before cross-linking. The oxygen-free solution was injected into a square container (30 × 15 mm) in the dark and was placed in a 70 °C oven for 12 hours to conduct the radical-copolymerization for obtaining the hydrogel. Tb^{3+}-containing and co-doped hydrogels were prepared with a similar method.

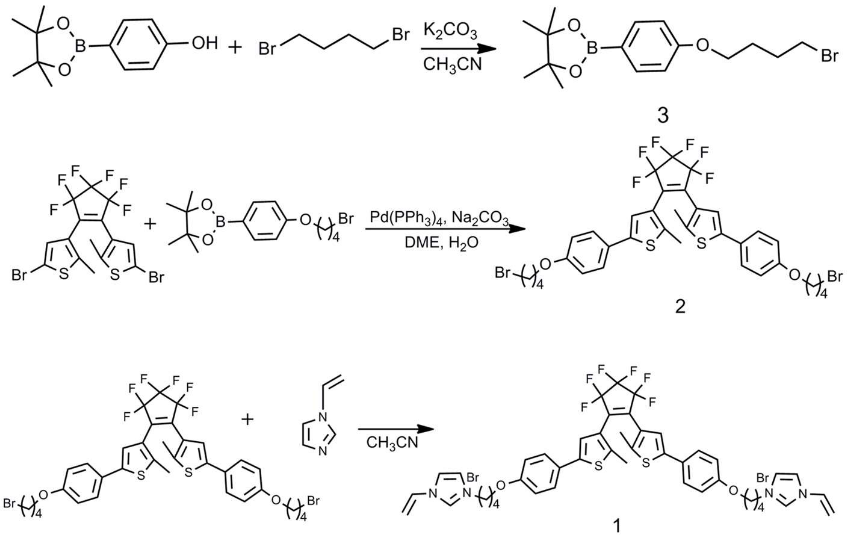

Scheme S1. Synthetic route of compound **1**.

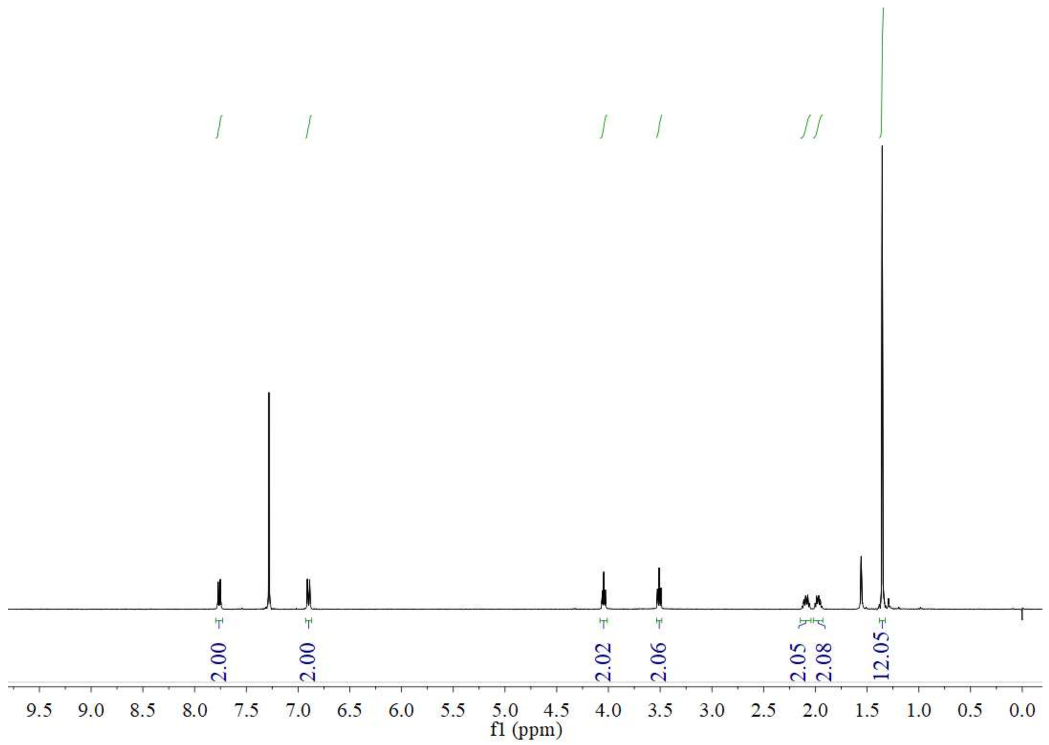

Figure S1. ^{1}H NMR spectrum of compound **3** ($CDCl_3$, 400 MHz, 25 °C).

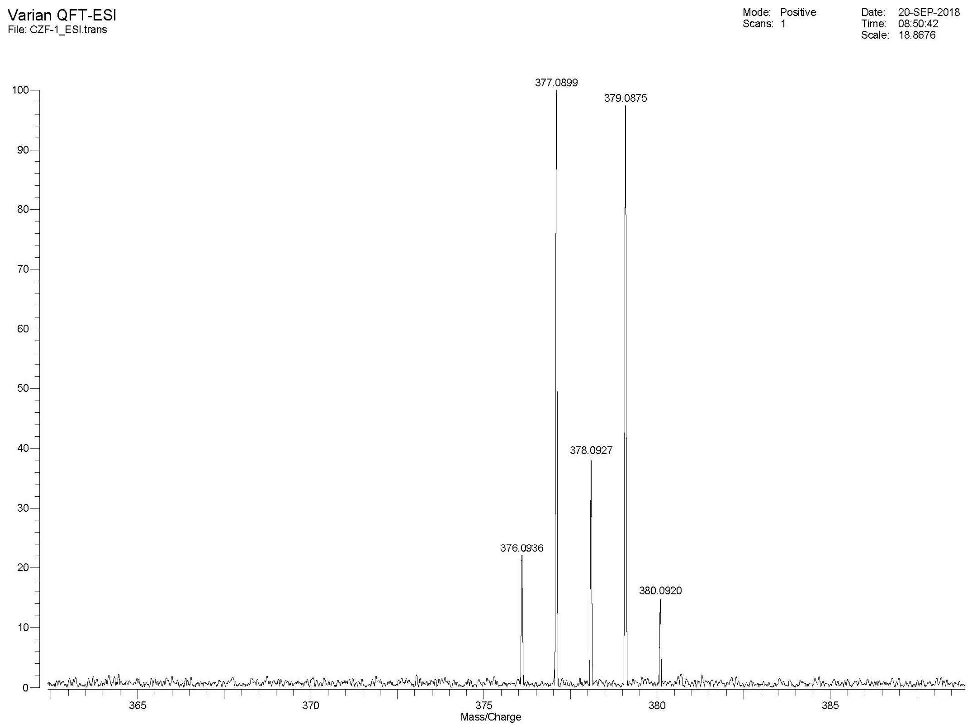

Figure S2. HRMS spectrum of compound **3.**

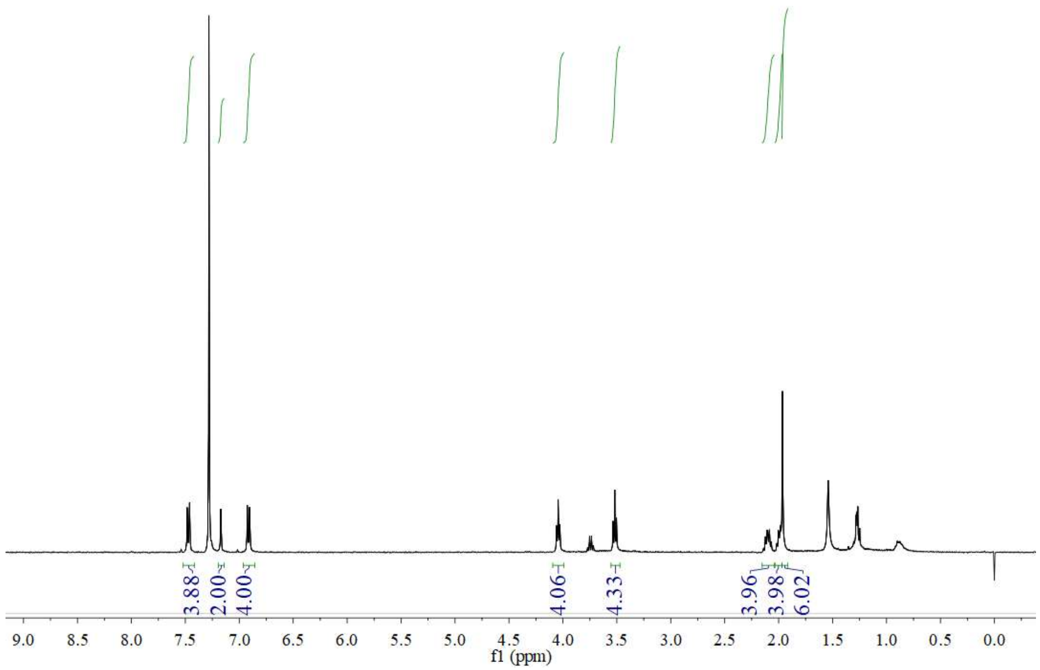

Figure S3. ^{1}H NMR spectrum of compound **2** ($CDCl_3$, 400 MHz, 25 °C).

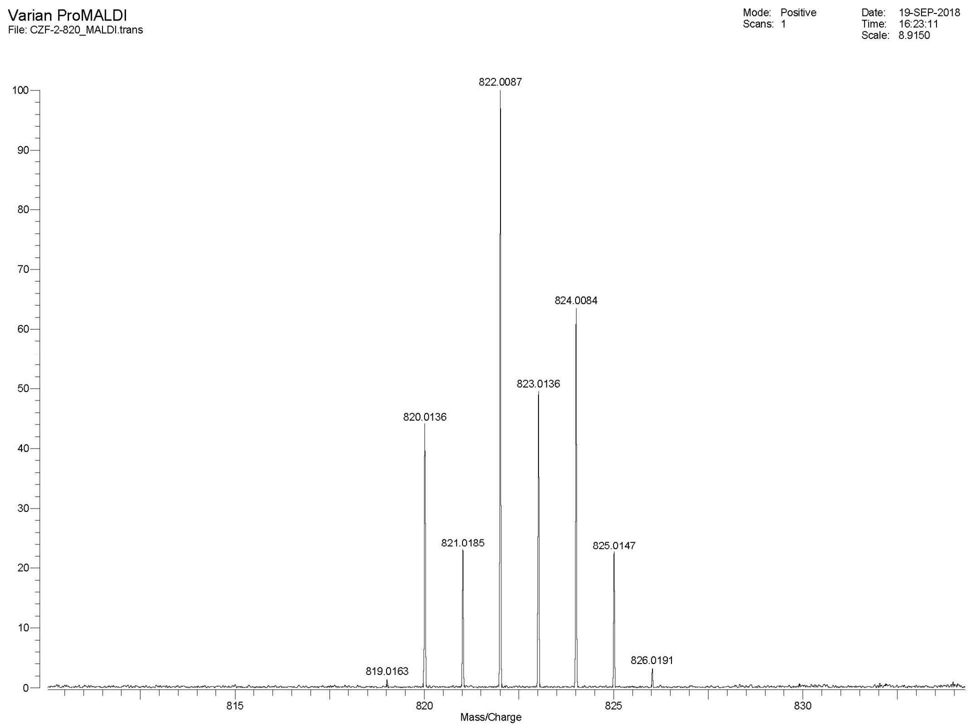

Figure S4. HRMS spectrum of compound **2.**

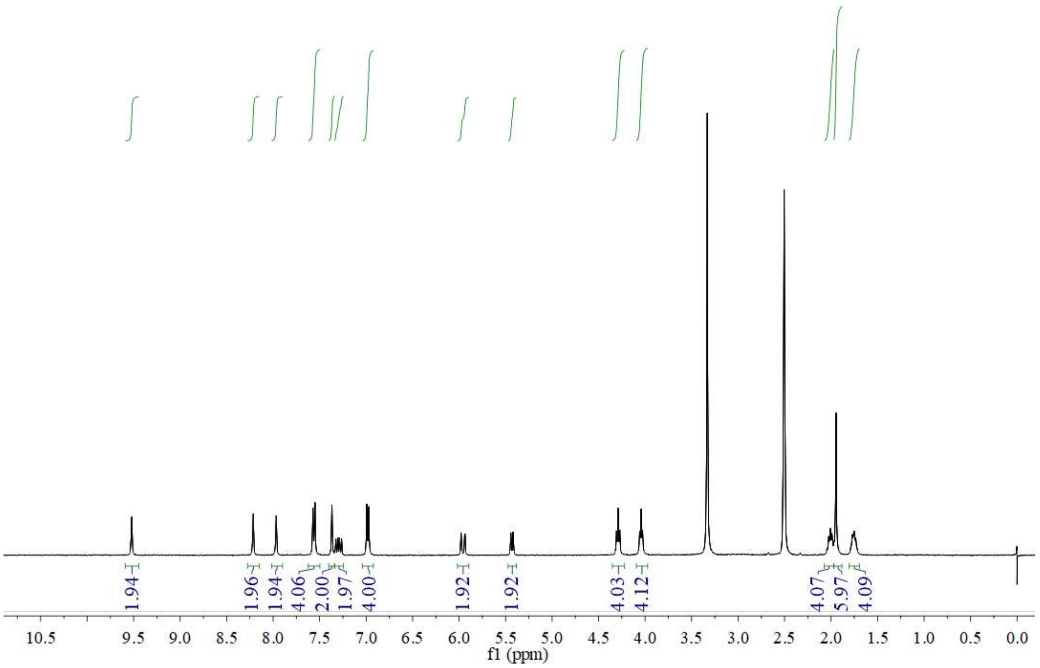

Figure S5. ^{1}H NMR spectrum of compound **1** (DMSO-d_6, 400 MHz, 25 °C).

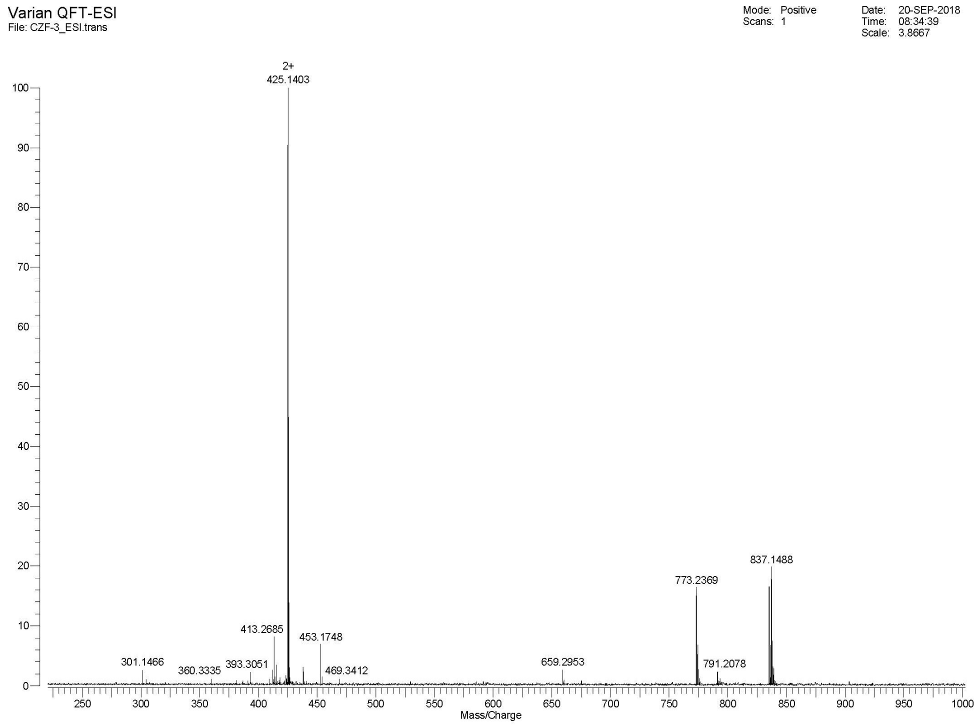

Figure S6. HRMS spectrum of compound **1.**

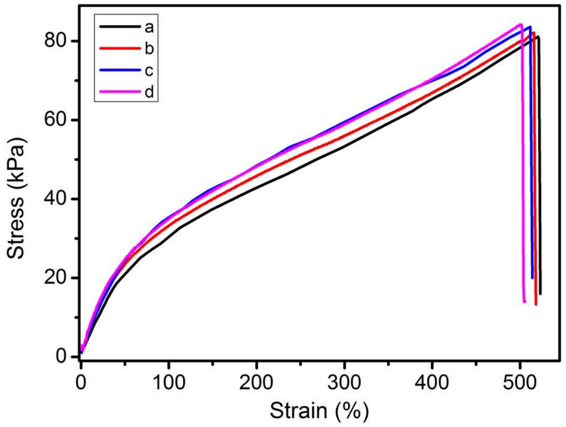

Figure S7. Tensile stress–strain curves of the Tb^{3+}-containing hydrogel (a) before and (b) after 300 nm UV light irradiation; Tb^{3+}/Eu^{3+} co-doped hydrogel (c) before and (d) after 300 nm UV light irradiation.

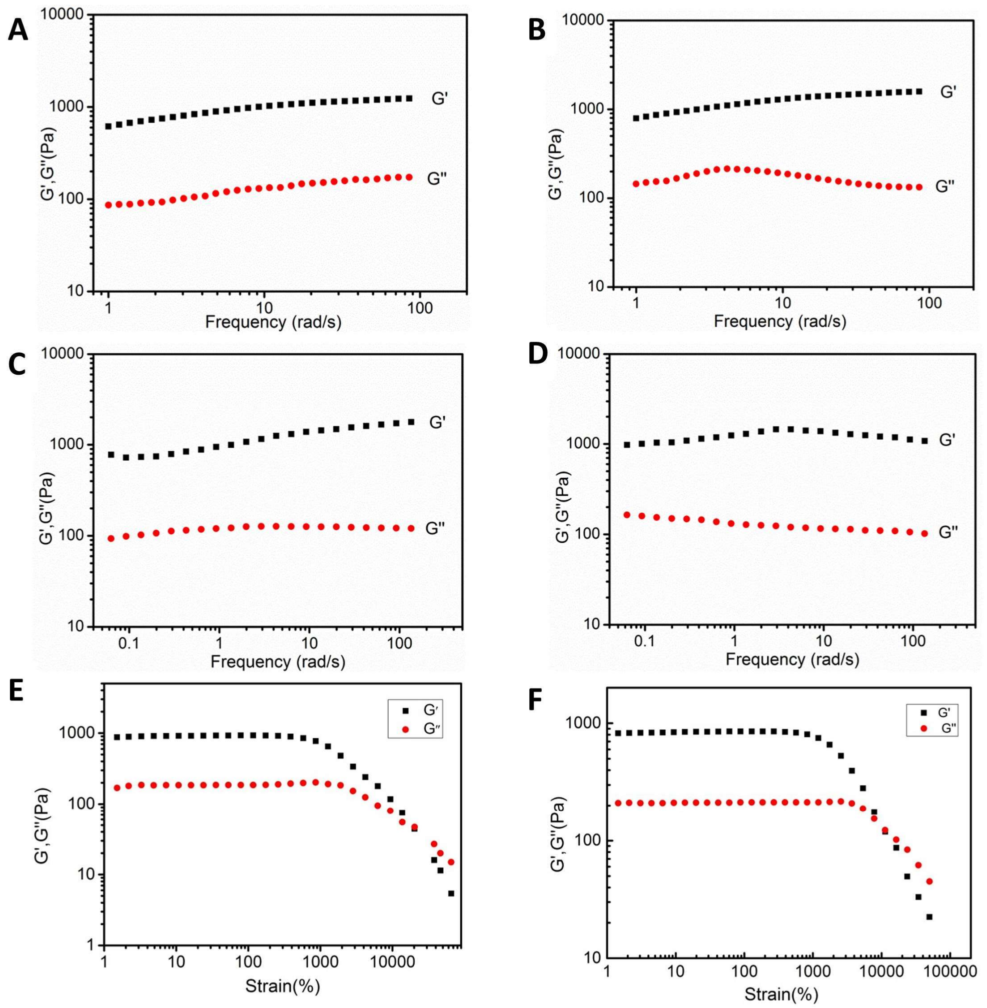

Figure S8. Frequency (ω) sweep tests at ω = 0.01 - 100 rad s^{-1} and strain (γ) = 1.0% for Tb^{3+}-containing hydrogel (A) before and (B) after 300 nm UV light irradiation; Tb^{3+}/Eu^{3+} co-doped hydrogel (C) before and (D) after 300 nm UV light irradiation at 25 °C. Strain sweep tests of Tb^{3+}-containing (E) and Tb^{3+}/Eu^{3+} co-doped hydrogel (F) hydrogel at γ = 0.1 - 100000% and ω = 1.0 Hz.

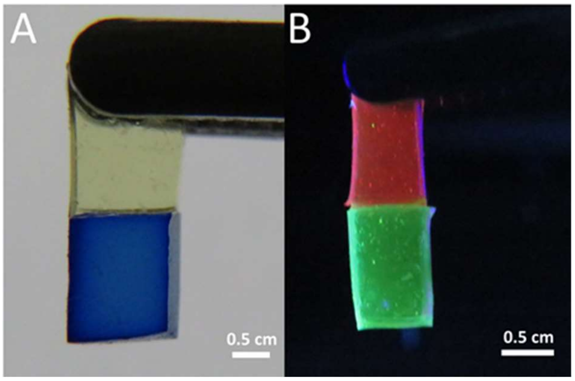

Figure S9. (A) Self-healing (re-annealing of Eu^{3+}-containing hydrogel before and after irradiation with 300 nm UV light) and (B) hetero-healing (cutting and reconnection of Eu^{3+}-containing and Tb^{3+}-containing hydrogels) behaviors.

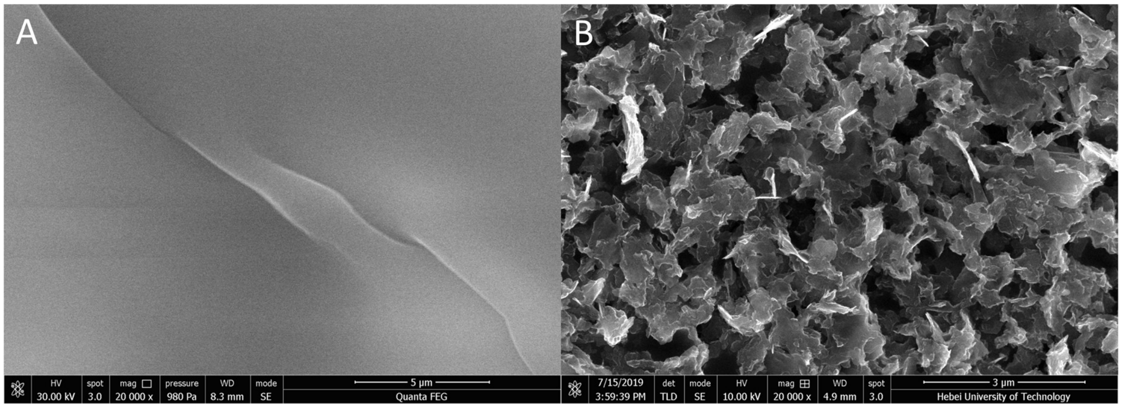

Figure S10. SEM images of (A) Eu^{3+}-containing hydrogel and (B) freeze-dried Eu^{3+}-containing hydrogel.

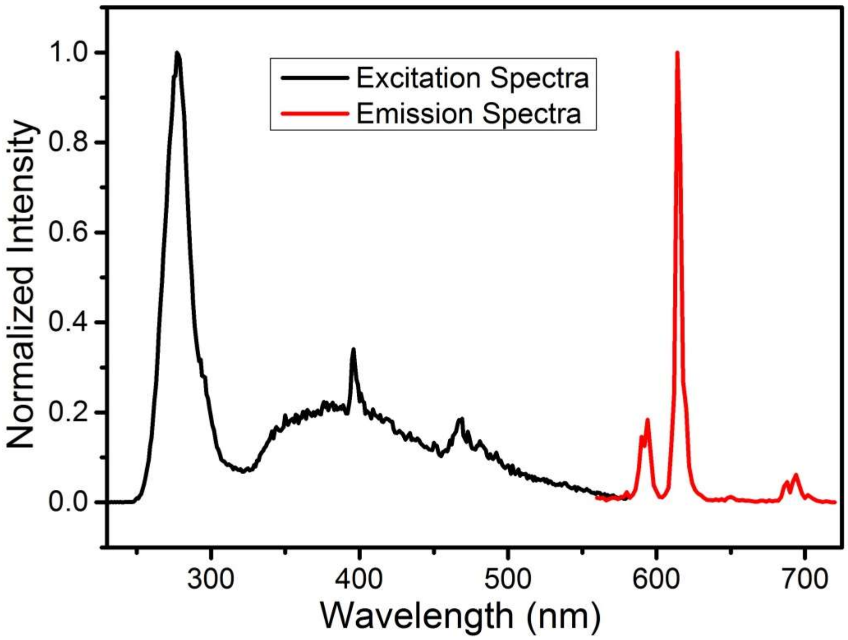

Figure S11. Normalized excitation (black) and emission (red) spectra of Eu^{3+}-containing hydrogel (excited at 280 nm and monitored at 615 nm).

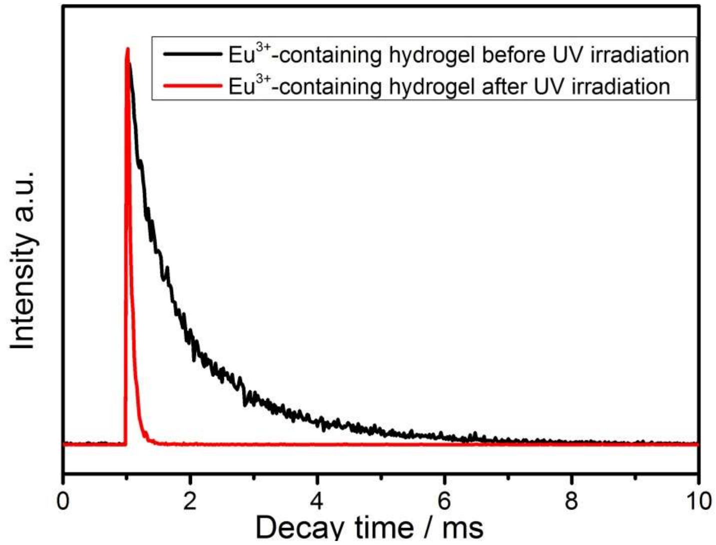

Figure S12. Luminescence decay curves of Eu^{3+}-containing hydrogel before (black line) and after (red line) irradiation with 300 nm light (excited at 280 nm and monitored at 615 nm).

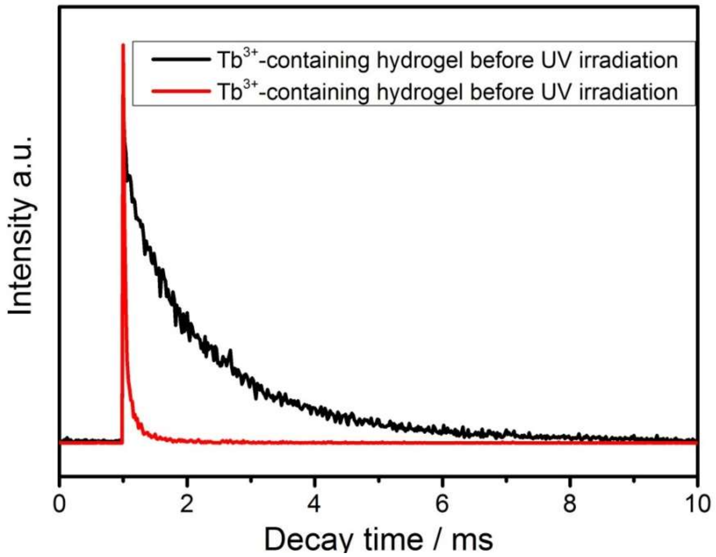

Figure S13. Luminescence decay curves of Tb^{3+}-containing hydrogel before (black line) and after (red line) irradiation with 300 nm light (excited at 280 nm and monitored at 543 nm).

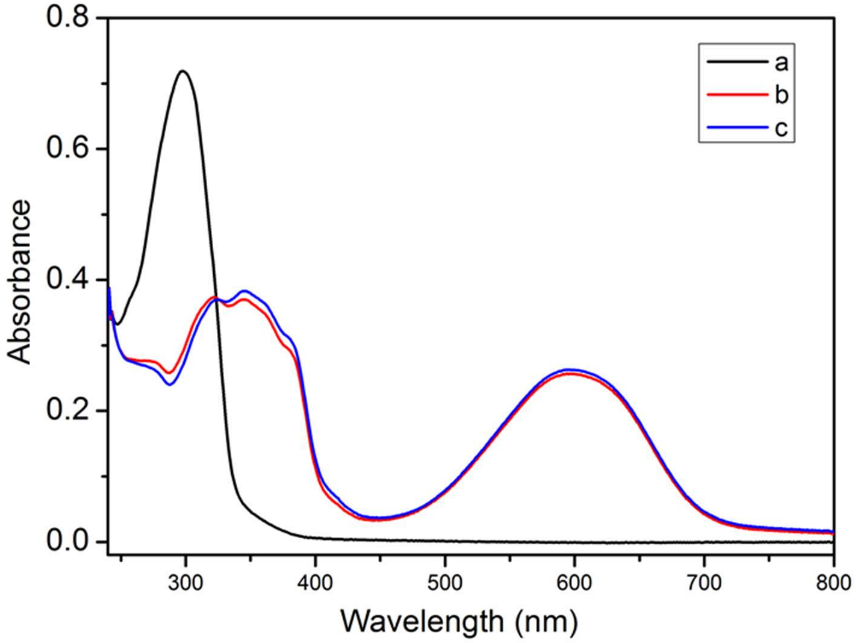

Figure S14. UV–Vis spectral of OF-**1** aqueous solution (2.0×10^{-5} M) before (a) and after (b) irradiated with 300 nm UV light for 60s, and further placed under daylight for 1 month (c).

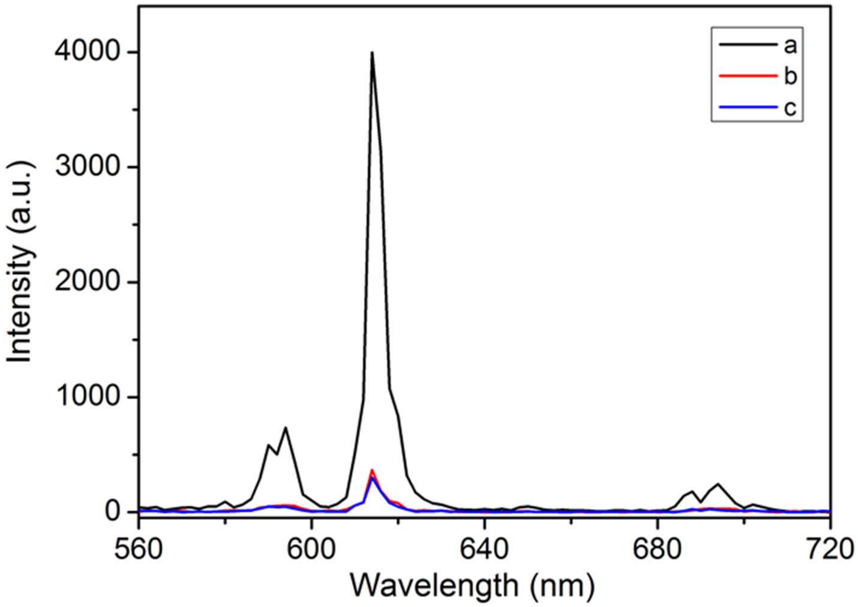

Figure S15. Luminescence emission spectral of Eu^{3+}-containing hydrogel (a) before and (b) after irradiated with 300 nm UV light for 60s, and (c) further placed under daylight for 1 month.

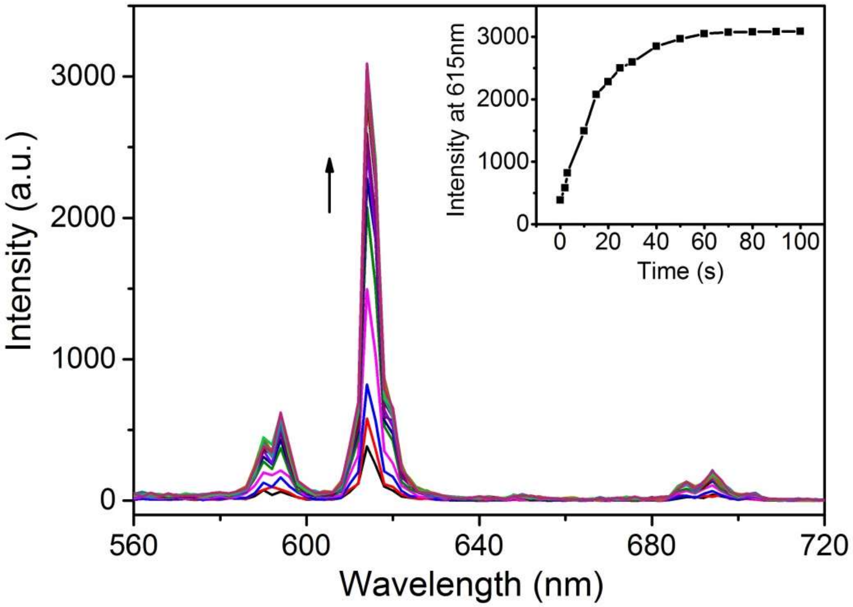

Figure S16. Luminescence emission spectral intensity and emission intensity changes (inset) at 615 nm for Eu^{3+}-containing hydrogel upon irradiation with visible light (> 450 nm).

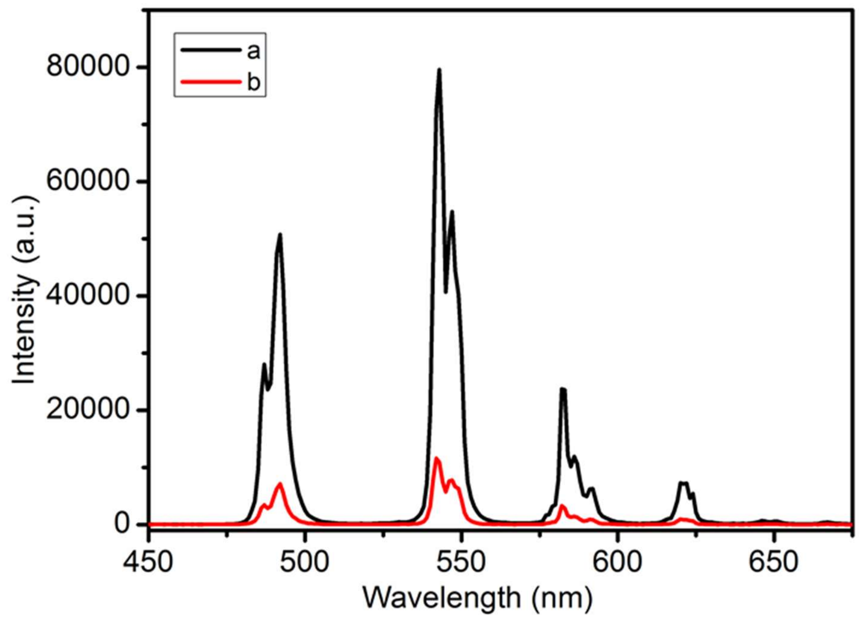

Figure S17. Luminescence emission spectra of Tb^{3+}-containing hydrogels (a) before and (b) after 300 nm UV light irradiation for 60 seconds (λ_{ex} = 280 nm).

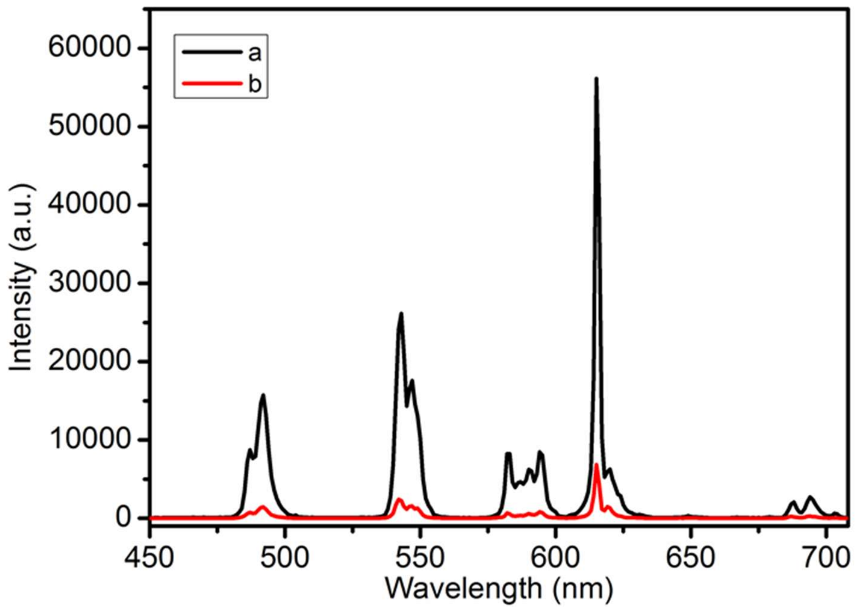

Figure S18. Luminescence emission spectra of Tb^{3+}/Eu^{3+} co-doped (Tb^{3+}/Eu^{3+} = 1:1) hydrogels (a) before and (b) after 300 nm UV light irradiation for 60 seconds (λ_{ex} = 280 nm).

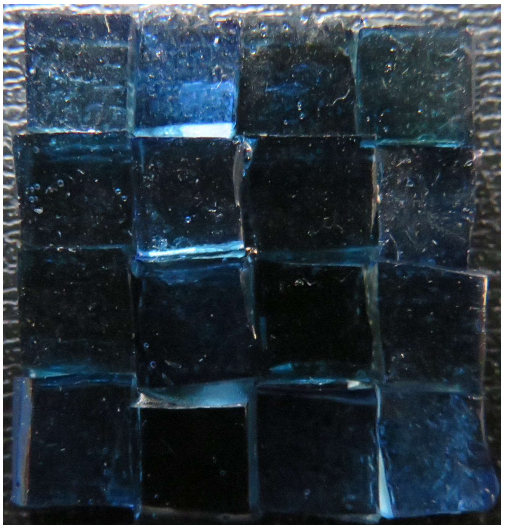

Figure S19. Photographs of a pattern (**Code A**) after irradiated with 300 nm UV light and then placed under daylight for 1 month.

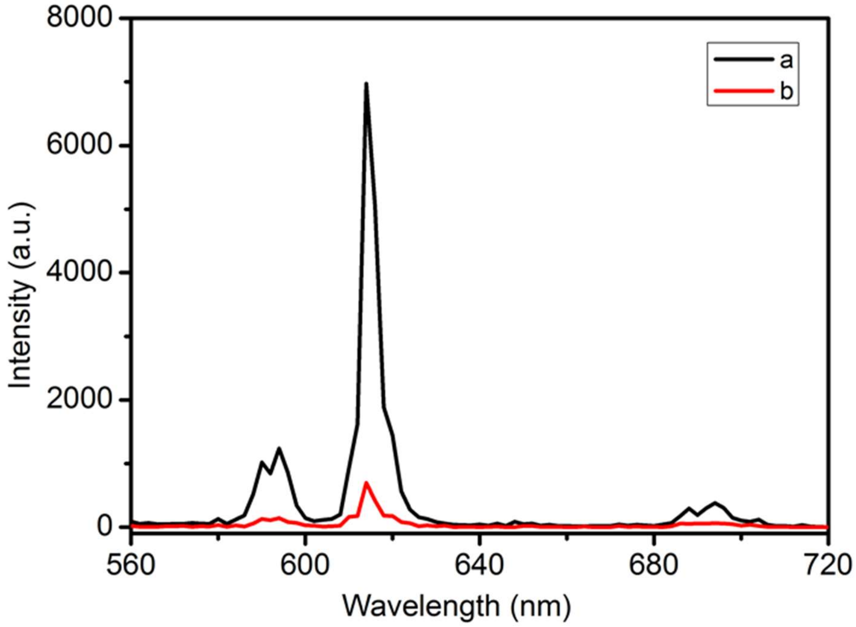

Figure S20. Luminescence emission spectra of Eu^{3+}-containing xerogel (a) before and (b) after 300 nm UV light irradiation for 60 seconds (λ_{ex} = 280 nm).

Table S1. Luminescence quantum efficiency (Φ), lifetime (τ) and number of coordinated water molecules (q) to Ln. τ_D and τ_H represent the luminescence lifetime determined in D_2O and H_2O, respectively.

Sample	Φ (%)	τ_H (μs)	τ_D (μs)	q
Eu^{3+}-containing hydrogel before UV irradiation	12.3	872	1554	0.30
Eu^{3+}-containing hydrogel after UV irradiation	0.6	68		
Tb^{3+}-containing hydrogel before UV irradiation	18.2	1260	1433	0.18
Tb^{3+}-containing hydrogel after UV irradiation	1.8	129		

Supporting videos

Movie S1: This movie shows the scanning of 3D **Code A** by a smart phone under daylight. The hydrogel blocks with various emission colors were placed on a substrate and contacted to construct the information pattern (24 mm × 24 mm × 2 mm), and then scanned by the smartphone under daylight. The emitting colors were invisible. As a consequence, the code was not recognized by the application software.

Movie S2: This movie shows the scanning of 3D **Code A** by a smart phone under UV lamp. **Code A** was exposed under a commercial UV lamp (254 nm) and scanned by the smartphone. The basic luminescent red, green, and yellow colors of Eu^{3+}-containing, Tb^{3+}-containing and co-doped hydrogels became visible and easily recognized by the software, allowing read out of the information (**Info A**).

Movie S3: This movie shows the scanning of 3D **Code A** after irradiation with UV light (300 nm). **Code A** was irradiated with a 300W Xe lamp under specific wavelength (300 nm) for 2 min, and then exposed under commercial UV lamp and scanned by the smartphone. The array turned to dark blue accompanied with the disappearance of the luminescent pattern. As a result, the coding information became invisible and cannot be read out.

Movie S4: **Code A** was irradiated with a 300W Xe lamp under specific wavelength (300 nm) for 2 min, and then exposed under daylight for one month. Then exposing the pattern under commercial 254 nm UV lamp and scanned by the smartphone. As a result, the coding information was still unreadable.

Movie S5: This movie shows the scanning of 3D **Code A** after 10 consecutive cycles of alternating exposure to UV and visible light. **Code A** was exposed under 300 nm UV light for 2 min, and then irradiated with visible light (>450 nm) for 2 min. Repeating this process for 10 times, and then scanning by the smartphone under commercial UV lamp were conducted. The information can be read out repeatedly without any hysteresis.

Movie S6: This movie shows the scanning of 3D **Code B** re-assembled from **Code A** under commercial UV lamp. We changed the position of hydrogel blocks in **Code A** to rebuild a new information pattern (**Code B**), loaded with another specific website (**info B**). Then, **Code B** was exposed under commercial UV lamp and scanned by the smartphone to read out the new information.
